# Improvement of Rice Husk/HDPE Bio-Composites Interfacial Properties by Silane Coupling Agent and Compatibilizer Complementary Modification

**DOI:** 10.3390/polym11121928

**Published:** 2019-11-22

**Authors:** Jingmeng Sun, Yao Pang, Yingni Yang, Junqi Zhao, Rongqi Xia, Yanchen Li, Yi Liu, Hongwu Guo

**Affiliations:** 1 MOE Key Laboratory of Wooden Material Science and Application, Beijing Forestry University, Beijing 100083, China; tulipsjm@163.com (J.S.); pangyao508@foxmail.com (Y.P.); yyingni_0922@163.com (Y.Y.); zhaojunqi77@163.com (J.Z.); xiarongqicn@163.com (R.X.); lyc100083@163.com (Y.L.); 2 Beijing Key Laboratory of Wood Science and Engineering, Beijing Forestry University, Beijing 100083, China; 3 MOE Engineering Research Center of Forestry Biomass Materials and Bioenergy, Beijing Forestry University, Beijing 100083, China

**Keywords:** rice husk, high-density polyethylene, bio-composite, interface modification

## Abstract

Composites using agricultural and forestry residues as raw materials with potentially high-performance, multifunctional and biodegradable ecological advantages, are viewed as very promising for new-generation lightweight and low-cost bio-based sustainable building materials. At present, the research on wood-plastic composite materials is relatively mature. However, it is still a challenge to effectively use other biomass and improve the interface of the high-polymer compound system. Herein, we proposed a simple and effective method to enhance the interfacial adhesion properties of rice husk fibre and High Density Polyethylene (HDPE) composites by the silane coupling agent KH-550 and compatibilizer Maleic anhydride grafted polyethylene (MAPE) with complementary modification. It was found that the coupling agent KH-550 cross-linked with the hydroxyl group on the husk fibre surface and solidified with the high polymer by –NH–, –C=O– functional group generation. Compatibilizer MAPE strengthened the two phases by covalently bonding with an ester linkage and lowered the roughness of the cross-section of the composites. Meanwhile the modification enhanced the dispersibility, and mechanical properties of the husk-high polymer compound system, the bending and flexural strength were improved by 11.5% and 28.9% with KH-550, and MAPE added, respectively. The flexural strength of the composites increased by 40.7% after complementary modification. Furthermore, the complementary modification treatment reduced the hydrophilic hydroxyl groups and increased the molecular chain to improve the water-resistance, elastic modulus and toughness of the composite. This study prepared a bio-composite, which is expected to expand the use of agricultural and forestry residues as an extension of wood-plastic composites.

## 1. Introduction

Composites using agricultural and forestry residues as raw materials with potentially high-performance, multifunctional and biodegradable ecological advantages, are viewed as very promising for new-generation lightweight and low-cost bio-based sustainable building materials. Rice is the main human crop, with a large area and giving rice husk as a by-product of rice production [1], the annual output of it is large but most of it is burned by producers. However, its main components are similar to wood with constituents such as cellulose, hemicellulose, lignin and silica [2]; the current use of husk is mainly in situ burning, feed or activated carbon, materialization [3,4,5], energy [6,7,8]. Due to a large amount of cellulose in the rice husk, rice husk may be a potential substitute for alternative wood, used to make composite materials as a complement to wood plastics; especially automotive, packaging and construction applications have broad application prospects.

However, the outer surface of the rice husk contains many ordered conical protrusions. Its main components are lignin and silica, which form a natural protective layer in the form of a silicon-fibre membrane [9]. The silicon in husks is mainly distributed in the conical protrusions of rice husks [10], which makes it hard to bond [11,12,13,14,15]. The rice husk has high hydrophilicity, but the high polymer material has poor hydrophilicity, which makes it difficult to polymerize with fibres. The reason for this difference is the number of hydroxyl groups on the surface of the material. However, the interface between rice husk and polymer matrix directly affects the comprehensive performance of the composite, thus improving the interfacial adhesion between two phrases and has become a hot research topic.

A large amount of researches have been conducted on the modification of natural fibres and their subsequent characterization in composites, which are mainly carried out by pre-treatment of plant fiber raw materials [16,17], adding interfacial modification aids during the preparation to acetylation [18], silanes treatment [19] and Malay treatment [20] to enhance the fiber/matrix interfacial adhesion strength [21,22].

Pre-treatment of plant fibres can remove free water and some bound water in the rice husk fibres and evaporate to reduce the hydroxyl content of the fibre surface [23]. Furthermore, it can effectively dissolve and remove low molecular substances in plant fibres [24], and reduce the hemicellulose and lignin content, resulting in an increased surface roughness of rice husk, which increases hydroxyl activity inside the fibre [25], and enhances the mechanical bond strength between polymer matrix and fibre [9,26].

The acetylation of the fibre can make the strongly polar hydroxyl group reacted with the weakly polar acyl group to form an ester compound, which can promote the dispersion of the fibre in a matrix such as plastic or resin [27]. It was found that a small amount of maleic anhydride grafted polyethylene (MAPE) can significantly heighten the mechanical properties of bamboo fibre/HDPE composites. Furthermore, the effect was better than with coupling agent [28,29].

In the modification of the interface between plant fibre and high polymer, chemical modification has received extensive attention for its advantages of simplicity and efficiency. Currently, more modifiers are used, such as silane coupling agents and compatibilizers. However, the current researches on modification methods are relatively simple, and the comparison of modification methods is less involved. In this work, it was attempted to modify rice husk and high-density polyethylene with different modifiers in order to prepare an ecological rice-plastic composite with good performance. The resource utilization of waste plastics and agricultural wastes provides a new way to reduce the consumption of timber resources. Moreover, it helps to promote resource conservation and recycling, which has an excellent ecological benefit.

## 2. Materials and Methods

### 2.1. Materials

Rice husks (RH) were obtained from the threshing of rice (Xu 91069) grown in Xuzhou, Jiangsu province, China, and the composition and content are shown in Table 1.

High-density polyethylene (HDPE) was provided by Daqing Oil Field Co. (Heilongjiang, China). Calcium carbonate (CaCO_3_, 1250 mesh) was supplied by Shanghai Macklin Biochemical Co., Ltd. (Shanghai, China). γ-Aminopropyltriethoxysilane (KH-550) was purchased from Nanjing Chuangshi Chemical Additive Co, Ltd. (Nanjing, China). Maleic anhydride grafted polyethylene (MAPE, 0.8%–1.2%, graft ratio) was purchased from Shengbang Engineering plastic raw materials business department (Dongguan, China).

### 2.2. Preparation of Composite Materials

The rice husk powder was pre-treated by milling. The rice husk was milled and pulverized by grinding discs, repeatedly milled 5–6 times and sieved to obtain 60 mesh rice hull powder. The pre-treated rice husk powder was dried to constant weight in an electric blast drying oven (DHG-9240, Heng Scientific, Shanghai, China) at a temperature of 103 ± 2 °C. Then the mixture (Table 2) was placed in a high-speed mixer (SHR-25A, Yongli Extrusion, Zhangjiagang, China) with low-speed stirring and high speed stirring for 10 min each. Compounding was achieved by a twin-screw extruder (KS-20, Kasun, Jiangsu, China). The temperature of each section of the extruder was set to 100 °C/125 °C/150 °C/130 °C/130 °C. Then, the extruded materials were cooled and granulated into pellets of length 2–5 mm by using a granulator (LQ-60, Kefei, Jiangsu, China). A hot press (BY-30, Xinxieli, Suzhou, China) was used to press the plates. The hot-pressing temperature was 160 °C, the pressure was 4 MPa, and the hot-pressing time was 9 min. Subsequently, cold-pressing (CGYJ-100, Cango, Shijiazhuang, China) was performed. The cold press pressure was 4 MPa, and the time was 5 min. Finally, a rice-plastic composite sheet with a size of 270 mm × 270 mm × 3 mm was produced.

### 2.3. Bending Properties

The bending strength (MOR) and bending modulus (MOE) of the rice-plastic composites were evaluated by a Microcomputer control electronic universal ability test machine (CMT6103, MTS, China). The samples with dimensions of 70 mm × 25 mm × 3 mm were tested in three-point bending mode with a loading speed of 5 mm/min. Moreover, six samples were tested in each group. After the maximum load value was reached, the bending strength and bending modulus of each sample were recorded and averaged. The standard error calculation method is as shown in Equation (1).

(1) $$SE=\sqrt{\frac{N}{N-1}\sum_{i=1}^{n}{\left(X_{i}-M\right)}^{2}}$$

where *N* is the number of samples, X_i_ is the measure of the mechanical properties of each sample, *M* is the average of the mechanical properties of the sample.

### 2.4. ATR-FTIR Analysis

The rice husk/HDPE composites were milled into powder and dried. The composites were analysed by an FTIR spectrometer (Spectrum GX, Perkin-Elmer, Waltham, MA, USA). KBr disks measurement was used, and the samples were recorded in the range 4000–400 cm^−1^ with a resolution of 2 cm^−1^ and 32 scans.

### 2.5. Thermal Properties

The thermal stability of husks and husks/HDPE composites were determined using a TA Instruments (TGA2050, TA, New Castle, DE, USA). The specimens were scanned from room temperature to 900 °C at a heating rate of 10 °C /min in the presence of nitrogen.

### 2.6. Dynamic Viscoelasticity

The dynamic viscoelasticity of the composites was evaluated by a dynamic mechanical analyser (Q800, TA Instruments, Baker, FL, USA). The samples with dimensions of 50 mm × 10 mm × 3 mm were tested in three-point bending mode with a heating rate of 5 °C /min from –50 to 150 °C at 5 Hz to obtain the values for storage modulus (E′), loss modulus (E″) and loss factor (tan δ). Moreover, the amplitude was 15 μm, the load 7.5 N, and the test mode a single cantilever beam mode.

### 2.7. Scanning Electron Microscopy (SEM) Analysis

The rice husk/HDPE composite sample was dried in advance. The surface and section of the samples were intercepted and fixed on the sample holder with conductive adhesive. The surface and section of the composites were observed by a scanning electron microscopy (JSM-7001F, JEOL, Tokyo, Japan). Before analysis, the samples were coated with Au using DC Sputtering. The SEM operated at an accelerating voltage of 20 kV, and the resolution was 1.2 nm/15 kV.

### 2.8. Surface Contact Angle Measurement

Contact angle measurements were performed with an optical contact angle apparatus (Oca 20, Dataphysics, Filderstadt, Germany). The test liquids used distilled water. Sessile droplets of liquids (3 μL) were placed on the composite surface (20 mm ×10 mm ×3 mm), the right and left angles of the drops on the surface were collected at intervals of 1s for a total duration of 60 s, five drops per sample were captured, and the average of angles was calculated.

## 3. Results and Discussions

### 3.1. Effect of Interface Modification on Bending Strength

**Figure 1:**
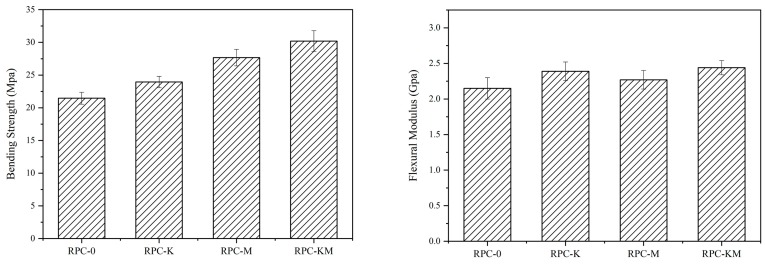
The bending strength and bending modulus of the composites without or with modifiers.

The bending strength and bending modulus of the composites without or with modifiers are shown in Figure 1. After adding the modifier, the bending properties and elastic modulus of the composites were improved. Modification enhanced the dispersibility and mechanical properties of the husk-high polymer compound system, the bending strength of composite with added KH-550 (RPC-K) increased by 2.47 MPa (11.5%), and that of the composite with added MAPE (RPC-M) increased by 6.21 MPa (28.9%). This demonstrated that the interfacial modifier enhanced the bonding of the rice husk fibre to the polymer and improved the mechanical properties of the composite. Additionally, the bending strength of composite with two added modifiers (RPC-KM) increased by 8.73 MPa (40.7%), which was higher than that of the material adding a simple modifier, when compared with the composites without added modifiers.

Flexural modulus refers to the ability of a material to resist bending deformation within the elastic limit. The larger the value, the less resistive the ability of the material to resist bending deformation within the elastic limit. It can be inferred that the toughness of the modified composite material was enhanced. It is speculated that the modifier may be longer when the modifier is combined with the rice hull and the high polymer, and the toughness of the composite material is enhanced. The reason for the difference in improvement effects may be that the molecular weight of KH550 is 221, and the molecular weight of maleic anhydride is 98.06, which is much smaller than the silane coupling agent. However, the co-modification effect of the two modifiers on the composite material is not as good as the modification effect of the silane coupling agent on the composite material. On the one hand, it may be caused by a chemical reaction between the modifiers, which causes a certain degree of loss. When the two are connected, the molecular weight of maleic anhydride is small, and the improvement of the composite material is mainly dominated by the silane coupling agent.

### 3.2. Effect of Interface Modification on Chemical Structure

**Figure 2:**
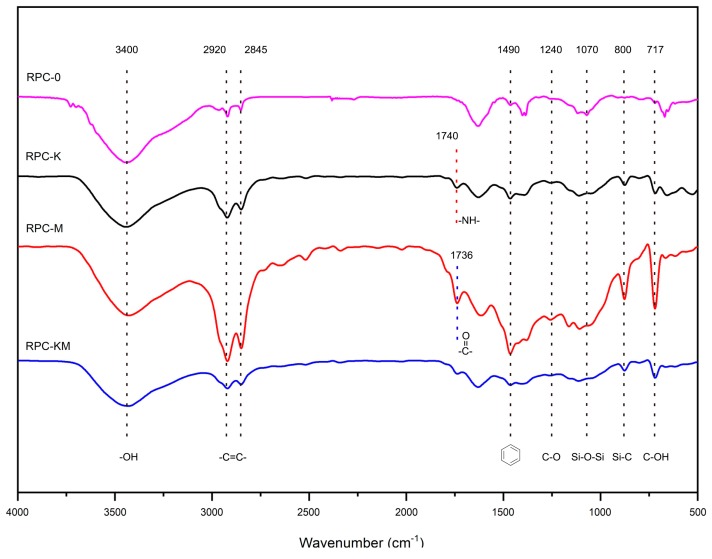
The infrared spectrum of composites without modifiers and with modifiers.

Figure 2 shows the infrared spectrum of the composite without modifiers and with modifiers. It can be seen from the analysis that 2920 cm^−1^ and 2844 cm^−1^ are the characteristic peaks of polyethylene [28,29]. While the stretching vibration of Si–O–Si at 1070 cm^−1^ and Si–C at 800 cm^−1^ are characteristic peaks of rice husk [30]. Moreover, 3400 cm^−1^, 1240 cm^−1^, 1166 cm^−1^ and 717 cm^−1^ are characteristic peaks of cellulose and hemicellulose in plant fibres [31,32,33], while the peak at 3400 cm^−1^ may be attributed to the –OH stretching vibration of cellulose and hemicellulose [34]. The stretching vibration of the aromatic ring at 1490 cm^−1^ and the characteristic peak of the ester bond at 1736 cm^−1^ are characteristic peaks of MAPE.

**Figure 3:**
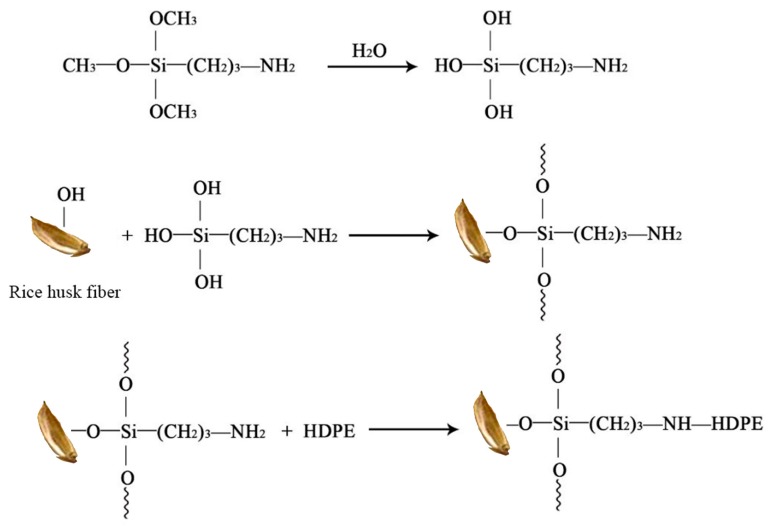
The reaction between silane coupling agent and rice husk fibre/HDPE.

The composite with the addition of the silane coupling agent KH-550 showed an absorption peak at 1740 cm^−1^ which may due to the presence of the –NH– group [35], indicating that KH-550 and fibres and polyethylene reacted. The silane coupling agent could serve as a bridge between the interface between the inorganic substance and the organic substance, and the reaction mechanism can be represented in Figure 3. The –OC_2_H_5_ group of KH-550, which was connected to Si at one end, was hydrolysed in ethanol solution to form Si–OH containing oligosiloxane, which underwent a condensation reaction with hydroxyl groups on the surface of rice husk fibre during heat curing to form covalent bonding at the other end. Additionally, the other end had an active amino group that cured with the polymer [35,36,37].

**Figure 4:**
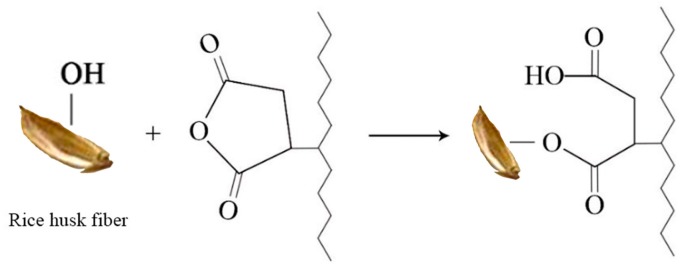
The reaction between rice husk fibre and MAPE.

In the spectrum of the composite with the compatibilizer MAPE added, the characteristic peak of carbonyl(–C=O–) stretching vibration at 1736 cm^−1^ indicated that the anhydride group in maleic anhydride was esterified with the free hydroxyl group in the fibre. The reaction principle of maleic anhydride grafted polyethylene and rice husk fibre is shown in Figure 4. The maleic anhydride group of MAPE was covalently bonded to the hydroxyl group of the fibre to form an ester bond [36], which reduced the surface energy of the fibre, while the non-polar portion (PE) of MAPE was compatible with the high-density polyethylene matrix which promoted the dispersion and compatibility of the fibres in the plastic matrix.

**Figure 5:**
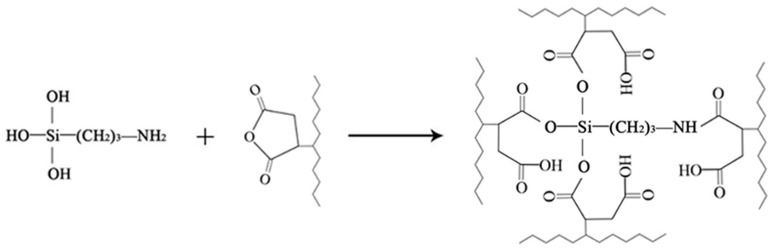
The reaction between silane coupling agent and MAPE.

Also, it can be noted that when the two compatibilizers were added, the peak intensity of each composite was different at the external bending vibration zone of –CH at 717 cm^−1^. The potential reason for the difference may be that the addition of the modifier increased the number of –CH groups, while the molecular structure of KH-550 and MAPE was different, so the number of groups was different such that the peak intensity of the composite showed a difference. At the same time, the peak intensity of the benzene ring at 1490 cm^−1^ was weakened. It was speculated that an esterification reaction between KH-550 and MAPE was carried out. The reaction principle of maleic anhydride grafted polyethylene and silane coupling agent is shown in Figure 5. The maleic anhydride group of MAPE was covalently linked to the hydroxyl group of KH-550 to form an ester bond, which opened the ring structure of MAPE. This also caused the internal loss of the modifier, so that the performance of the sample with two added compatibilizers did not meet the expected performance.

### 3.3. Effect of Interface Modification on Thermal Stability

**Figure 6:**
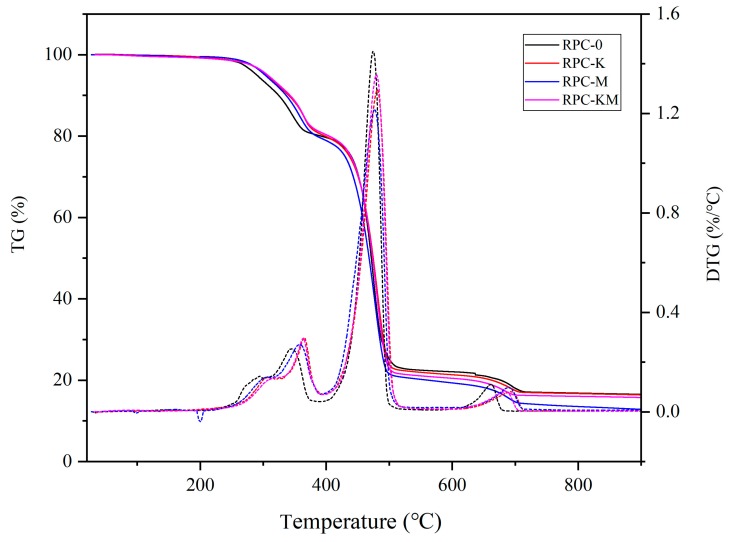
TG-DTG curve of composites without or with modifiers.

The thermogravimetric curve of the composite without addition or with modifiers is shown in Figure 6. Pyrolysis of composite materials consists of five steps. The first three steps are drying, transition and pyrolysis of rice husks. In the first step, the moisture in the rice husk is volatilized by heat, and the quality slightly decreases in the range of room temperature to 150 °C. Moreover, in the second step, rice husk depolymerises and vitrifies, and small molecular weight compounds are released in the range of 150–220 °C [38]. The rice husk components are pyrolyzed, and the volatiles mainly precipitated in the range of 220–400 °C. The thermal decomposition occurs at this step [39,40]. Rice husk fibre and the high-density polyethylene pyrolyze together in the range of 400–500 °C, and almost completely decompose. In the fifth step, thermally stable calcium carbonate pyrolyzes. After 750 °C, entering the carbonization stage, the curve tends to be gentle.

The first pyrolysis peak is the pyrolysis of rice husk. When the interface modifier was added, the pyrolysis peak gradually moves to the high temperature zone, indicating that the interfacial modifier enhanced the bonding between the two phases, which hindered the fibre. The rapid decomposition further indicated that the fibres and the high-density polyethylene matrix were tightly connected under the action of the interface modifier, and the thermal motion of the fibres was bound. It can also be seen from previous infrared analysis that the modifier formed a chemical bond with the rice husk and the high-density polyethylene. There are three states: connected to KH-550; connected to MAPE; a small amount connected to both, but in this latter case, the spacing between rice husk and high-density polyethylene became large, prone to pyrolysis, and could not improve the thermal stability of the composite effectively. When both modifiers were added, the modification effect on the interface of the composite was not a superposition effect but partial internal friction occurred between the two modifiers. The second pyrolysis peak was the co-decomposition of rice husk and high-density polyethylene. It can be seen that the rate of pyrolysis was reduced to some extent after the addition of modifier, further indicating that the modifier increased the binding between the two phases.

### 3.4. Effect of Interface Modification on Dynamic Thermomechanical Properties

**Figure 7:**
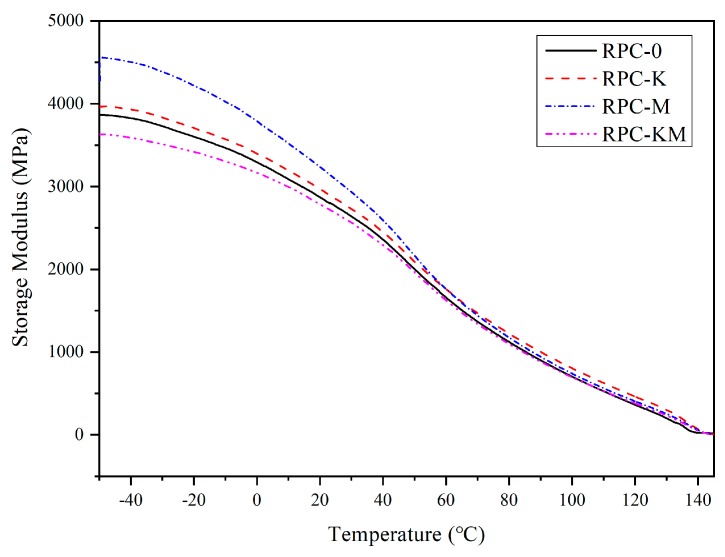
The storage modulus(E′) of rice husk/HDPE composites.

**Figure 8:**
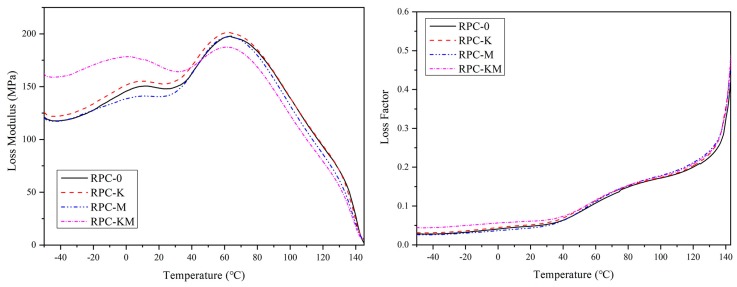
The loss modulus (E″) and loss factor (tanδ) of rice husk/HDPE composites.

The storage modulus (E′), loss modulus (E″) and loss factor tan δ of each composite material as a function of temperature are shown in Figure 7 and Figure 8.

The storage modulus can characterize the stiffness of the composite. The greater the stiffness, the lower the toughness of the composite. It can be seen from Figure 7 that after adding a single modifier, the storage modulus of the composite material was improved, and the initial storage modulus of RPC-M was increased by 500 MPa. However, the storage modulus of RPC-KM was lower than that of RPC-0. The composite material under low-temperature condition can be regarded as a rigid body. The increase of storage modulus may be due to the increase of matrix stiffness and the enhancement effect of rice husk fibre due to the addition of modifier, while the stress transfer between the two-phase interface became large. The storage modulus was related to the interface and toughness of the composite. The higher the storage modulus, the higher the interface bonding strength, and the smaller the toughness. After the addition of the modifier, the increase of the storage modulus indicated that the modifier improved the interfacial bonding between the rice husk and the high-density polyethylene, and it also made the molecular weight of the composite longer, improving the toughness of the composite materials to a certain extent. The RPC-M interface had the highest intensity. However, for the phenomenon that the interfacial strength of the composite material with the addition of two modifiers was decreased, it can be surmised that the molecular segment of the high-density polyethylene had changed due to the interaction of the silane coupling agent KH-550 and the compatibilizer MAPE. This improved the molecular motion ability and the segment mobility, thus exhibiting a weaker stiffness at low temperatures.

It can be seen from Figure 8 that the composite had two mechanical relaxation peaks in the tested temperature range, namely α- and β-transformations [41,42]. Relaxation peaks can reveal the molecular interactions between rice husk fibre and high-density polyethylene [43]. The β-transition process occurs near –10 °C, and the α-transition process appears around 60 °C. The reason for the relaxation peak was that the polymer was converted from a glassy state to a high-elastic state at this temperature, and the molecular segment movement was intensified. The composite materials of RPC-0, RPC-K and RPC-M have similar α- and β-transition temperatures, respectively, at 64 °C and 10 °C, respectively. While the transition temperature of RPC-KM shifted to the low-temperature region and had a higher relaxation peak height at 10 °C than the other composite materials, indicating that the molecular chain motion was restricted more at this time, and the molecular motion maximizes the energy loss. However, when α was converted, RPC-KM was lower than other composite materials, and the RPC-K peak was the largest. The relaxation peak was related to the mobility of the segments in the crystal in the composite, which may be due to the reorientation of defective regions in the crystal. It also can be seen from Figure 7 that the loss factor tan δ of the composite material increases with the increase of temperature, but the difference in the tan δ curves of each composite material was not obvious, indicating that the interfacial interaction forced between rice husk powder and polyethylene matrix was similar.

### 3.5. Effect of Interface Modification on Microstructure

**Figure 9:**
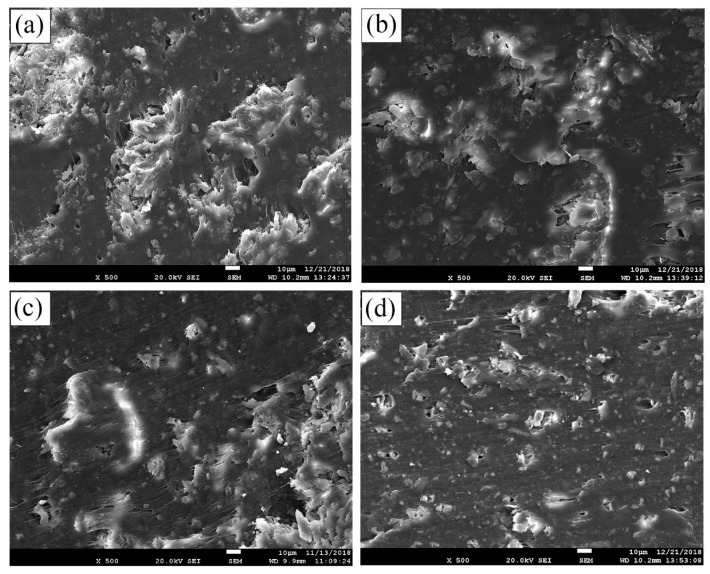
The cross-sectional micromorphology of composites without modifier (**a**) and with KH-550 (**b**), MAPE (**c**), KH-550+MAPE (**d**).

Observing the cross-sectional morphology of the composite can reflect the combination of rice husk and HDPE. The cross-section of RPC-0 (Figure 9a) was not uniform, even the rice husk particles were exposed, and there was also agglomeration. The interface between the two phases was clear, indicating that the rice husk was unevenly dispersed in the plastic matrix and could not be completely wrapped. Moreover, the interface was not well combined, resulting in local stress, which affected the mechanical properties and durability of the composite. When a single interfacial modifier was added, the surface of the composite was relatively flat, meanwhile the interface of RPC-K was flatter than that of RPC-M, which indicated that modifier ameliorated the dispersibility of rice husk in HDPE and reduced the local stress, resulting in water resistance of the composite, thus enhancing the mechanical strength and water resistance of the composite. When the two methods were simultaneously modified, the section of RPC-KM (Figure 9d) was relatively flat and homogeneous, the cracks and pores were small and sparse. This can be evidence that the rice husk can be more equally dispersed in HDPE, resulting in a good water-resistance and large storage modulus.

### 3.6. Effect of Interfacial Modification on Hydrophobic Properties

**Figure 10:**
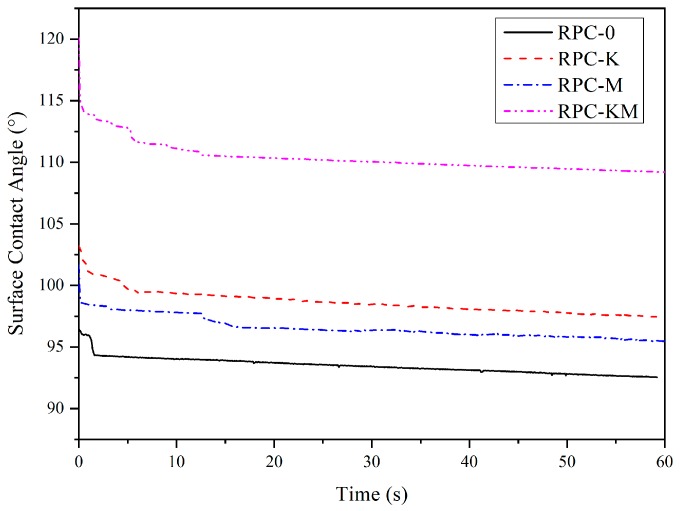
The dynamic contact angle of composites without or with modifiers.

The surface dynamic contact angle of each rice plastic composite is shown in Figure 10. The surface contact angle decreased slowly with time. The contact angle of each sample at the 60 s was higher than 90°, indicating that the surface of the composite was non-wetting and had certain hydrophobicity. Among them, the contact angle of RPC-KM was the largest, the contact angle of RPC-0 was the smallest, and the difference between the two was nearly 20°; while the contact angle of RPC-K was slightly higher than the contact angle of RPC-M.

By comparison, the addition of the interface modifier increased the surface contact angle of the composite, which in turn affected the surface wettability. From the analysis of electron microscopy and infrared, it can be inferred that the modifier improved the dispersibility of rice husks, reduced the agglomeration of rice husks, and formed a chemical bridge between rice husks and HDPE, reducing the surface hydroxyl groups of composites. Accordingly, the water-resistance of the composite was enhanced.

## 4. Conclusions

In summary, in order to promote the utilization of rice and agricultural and forestry residues and improve the comprehensive utilization rate, this study explored the possibility of combining rice husks with high molecular polyethylene to prepare bio-materials. In order to improve the interfacial adhesion properties of the composites, the modification effects of different modifiers on composites were investigated. FTIR, SEM and dynamic mechanical analysis confirmed that the interface modifiers reduced both the amount of hydroxyl groups on the surface and the surface energy of the fibre by chemical bridging between rice husks and HDPE, which promoted the dispersion and compatibility of the fibre in the plastic matrix, facilitated the interfacial bonding strength of the composite, and decreased the surface wettability of the composite. When the two complementarily modified the composite material, a reaction between KH-550 and MAPE occurred, which caused a certain modifier loss, but the overall modification effect was better than that of the single modifier. The flexural strength of the composite with added single interface modifier increased by 11.5%, while that of the composite containing the coupling agent and the compatibilizer increased by 40.7%. The study provided a green and simple method for the effective use of agricultural and forestry residues. The rice-plastic composite materials described in this study can be used in furniture building materials and plates, thus providing a way for the green utilization of building materials.

## Figures and Tables

**Table 1:** The composition and content of rice husk.

Composition	Cellulose	Hemicellulose	Lignin	Silica	Lipid	Moisture	Ash
Content (%)	30–35	15–25	20–26	20	0.7–1.3	5	15–25

**Table 2:** The experimental ratio of rice husk/HDPE composites.

Sample	RH (parts)	HDPE (parts)	KH-550 (parts)	MAPE (parts)	CaCO_3_ (parts)
RPC-0	40	60	-	-	10
RPC-K	40	60	5	-	10
RPC-M	40	60	-	5	10
RPC-KM	40	60	5	5	-
